# 766. "Assessing the impact of advanced vaccine education on pharmacy students’ knowledge, confidence, and preparedness in becoming future immunization providers: A National Study"

**DOI:** 10.1093/ofid/ofad500.827

**Published:** 2023-11-27

**Authors:** Ajit Johal

**Affiliations:** Immunize.io Health Association, Vancouver, British Columbia, Canada

## Abstract

**Background:**

With community pharmacists emerging as prominent immunization providers, there is an increased need for additional training in this area. The primary objective of this pre-post questionnaire study was to evaluate the impact of a comprehensive webinar on advanced vaccine education on the knowledge, confidence, and preparedness of undergraduate PharmD students across Canada.

**Methods:**

A 25-question, web-based survey was administered to PharmD students at seven Canadian pharmacy schools prior to and immediately after attending a comprehensive educational webinar from March 13th to April 7th, 2023. The pre- and post- survey data were analyzed using SPSS 26. A Friedman rank test was performed on questions which answers consisted of ordinal data.

**Results:**

In a group of **355** pharmacy students across Canada who attended our live educational webinars, **279** completed the pre-survey questionnaire, and **132** students completed the post-survey questionnaire. Pre-survey questionnaire results showed knowledge and confidence gaps in the areas of addressing vaccine hesitancy, immunization assessment, and recommended vaccinations. After the educational webinar event, students showed higher knowledge and confidence across all domains, and statistically significant improvements in critical areas pertaining to community vaccination practice. This highlights pedagogical changes which can be made to pharmacy school curriculums to better prepare students for future immunization practice.

**Conclusion:**

Considerable knowledge gaps exist for graduating community pharmacists in vaccine education. Pharmacy students will benefit from integrated education focusing on addressing vaccine hesitancy, immunization status assessment, and jurisdictional routine immunization programs. Communities may also benefit from frontline healthcare providers who are highly trained and empowered with an expanded scope of practice to address their lifespan vaccination needs.

**Disclosures:**

**Ajit Johal, BSP BCPP RPh**, CSL Seqirus: Advisor/Consultant|GSK: Honoraria|Merck: Honoraria|Pfizer: Honoraria

